# Clinical, Metabolic, and Behavioral Correlates of Nutritional Status in Chronic Heart Failure

**DOI:** 10.3390/nu18081269

**Published:** 2026-04-17

**Authors:** Katarzyna Lomper, Julia Buczkowska

**Affiliations:** 1 Division of Research Methodology, Department of Nursing, Faculty of Nursing and Midwifery, Wroclaw Medical University, 51-618 Wroclaw, Poland; 2 School of Health & Social Care, Edinburgh Napier University, Edinburgh EH11 4BN, UK; 3 Student’s Research Club, Department of Nursing, Faculty of Nursing and Midwifery, Wroclaw Medical University, 51-618 Wroclaw, Poland

**Keywords:** heart failure, malnutrition, Mini Nutritional Assessment, self-care behaviour, NT-proBNP, NYHA class, body mass index

## Abstract

Background: Heart failure (HF) is a chronic condition associated with frequent hospitalizations and impaired quality of life. Malnutrition is common in HF and is linked to adverse clinical outcomes, while self-care is an important component of HF management. This study aimed to examine the associations between nutritional status, self-care behaviors, and clinical characteristics in patients with chronic HF. Methods: A cross-sectional study was conducted among 100 hospitalized HF patients (mean age 75.9 ± 9.8 years; 63% men). Nutritional status was assessed using the Mini Nutritional Assessment (MNA), and self-care using the nine-item European Heart Failure Self-care Behaviour Scale (9-EHFScBS). Clinical variables included NYHA class, LVEF, comorbidities, BMI, and laboratory parameters. Comparative analyses and multivariate linear regression were performed. Results: Patients who were malnourished or at risk of malnutrition had significantly higher NT-proBNP levels (*p* = 0.004) and higher NYHA class (*p* = 0.002), whereas well-nourished individuals had significantly higher triglyceride levels (*p* = 0.032). Nutritional status was negatively associated with NYHA class and NT-proBNP, and positively associated with BMI. Among laboratory parameters, significant positive correlations were observed with hemoglobin, hematocrit, albumin, and triglyceride levels. In multivariate analysis, the following variables were independently associated with MNA score: self-care score (B = 0.083 per point), BMI (B = 0.368 per kg/m^2^), comorbidity burden (B = −0.401 per comorbidity), and NYHA class (NYHA III: B = −2.425; NYHA IV: B = −5.966, vs. NYHA II). Conclusions: In patients with chronic heart failure, nutritional status is associated with disease severity, metabolic parameters, comorbidity burden, BMI, and self-care behaviors. These findings support the importance of routine nutritional screening as part of comprehensive HF management.

## 1. Introduction

As a chronic, progressive syndrome with frequent exacerbations, heart failure (HF) contributes to poor patient outcomes and places a heavy burden on healthcare infrastructures globally. HF remains one of the most prevalent cardiovascular conditions worldwide [1] and is associated with high morbidity, frequent hospitalizations, and reduced quality of life [2,3]. Malnutrition in this group has been strongly linked to poorer clinical outcomes, including higher rehospitalization [4] rates and mortality [5]. The interplay between HF and malnutrition is pathophysiologically complex, driven by persistent inflammatory activation, splanchnic congestion leading to malabsorption, and neurohormonal and metabolic dysregulation [6]. HF is associated with a persistent pro-inflammatory and catabolic state that contributes to progressive tissue wasting and metabolic imbalance. Malnutrition, in turn, acts as a negative prognostic marker, exacerbating myocardial dysfunction, accelerating disease progression, and increasing the risk of adverse clinical outcomes. Chronic undernutrition may contribute to the onset of sarcopenia, a condition defined by the reduction in muscle mass, which consequently leads to diminished muscular strength, slower gait, and reduced overall physical activity [7]. This bidirectional relationship underscores the importance of early recognition and targeted management of nutritional deficiencies in those patients. Nutritional status assessment is therefore a critical component of comprehensive HF management, but HF guidelines provide only limited recommendations regarding the screening and management of nutritional disorders in this population [8]. At the same time, self-care plays a pivotal role in the long-term management of HF. Individuals with HF who engage in more effective self-care demonstrate improved quality of life, along with reduced mortality and readmission rates, compared to those with poorer self-care [9]. In addition to nutritional status, behavioral factors such as self-care are key determinants in shaping the clinical trajectory of HF patients. Given the interrelationship between nutritional status, self-care capacity, and disease severity, a detailed assessment of these factors may provide valuable insights into patient outcomes and guide targeted interventions in clinical practice. Incorporating structured nutritional evaluation into standard HF care may therefore represent an important step toward optimizing long-term outcomes.

## 2. Materials & Methods

This was a cross-sectional, observational study conducted among patients hospitalized in the Department of Cardiology. The primary objective of this study was to assess the associations between nutritional status, as measured by the Mini Nutritional Assessment (MNA), and selected clinical, biochemical, and behavioural parameters in patients with chronic heart failure. Additionally, we aimed to evaluate whether self-care behaviours and body mass index (BMI) independently predict nutritional status in this population.

The study population comprised 100 patients with a mean age of 75.9 ± 9.8 years. Patients were recruited consecutively during hospitalization. Inclusion criteria were as follows: adult patients (≥18 years) with a confirmed diagnosis of chronic heart failure, who were clinically stable at the time of assessment and provided informed consent to participate in the study. Exclusion criteria included a documented history of dementia, newly diagnosed HF, and lack of informed consent. All patients were admitted electively and were in a clinically stable condition at the time of assessment. None of the participants presented with signs or symptoms of acute heart failure decompensation. Anthropometric measurements were performed after clinical stabilization during hospitalization. Data were obtained through comprehensive review of medical records (comorbid conditions, current pharmacotherapy, NYHA functional class, and recent laboratory investigations including complete blood count, lipid profile, serum albumin, transferrin saturation [TSAT], and left ventricular ejection fraction), a self-administered sociodemographic questionnaire (age, sex, place of residence, marital status, occupational status, educational attainment, and monthly income), as well as two validated instruments: the Mini Nutritional Assessment (MNA) [10,11] and the nine-item European Heart Failure Self-care Behaviour Scale (9-EHFScBS) [12,13].

The MNA is a standardized screening tool for evaluating nutritional status, encompassing four domains: anthropometric indices, general assessment, dietary assessment, and self-perception. Scoring ranges from 24–30 points (normal nutritional status), 17–23 points (at risk of malnutrition), and <17 points (malnourished) [11]. In the present study, BMI was analyzed as a routinely available clinical parameter rather than as a precise marker of adiposity. Given the limitations of BMI in heart failure, particularly in the context of potential fluid retention, its interpretation was made in conjunction with nutritional status assessed by the MNA.

The 9-EHFScBS assesses the capacity of patients with heart failure to perform appropriate self-care behaviours. It consists of nine items, five of which pertain to routine self-care practices: daily weight monitoring, adherence to fluid restriction, compliance with prescribed pharmacotherapy, adherence to a low-sodium diet, and engagement in regular physical activity. The remaining items address recognition and management of symptoms associated with disease progression, including increasing dyspnoea, rapid weight gain, peripheral oedema, fatigue, and weakness. Each item is rated on a five-point Likert scale (1 = “completely agree” to 5 = “completely disagree”), yielding a total score from 9 to 45 points. Lower scores indicate more effective self-care, whereas higher scores reflect poorer self-care behaviours [12,13]. Self-care behaviors were measured using the nine-item European Heart Failure Self-care Behaviour Scale (9-EHFScBS). The original score (range: 9–45, with lower scores indicating better self-care) was reversed and standardized to a 0–100 scale according to the method proposed by Vellone et al., with higher scores indicating better self-care [14].

Statistical analysis was conducted using IBM SPSS Statistics, version 29. Due to the clinical nature of data collection, some laboratory parameters were available only for a subset of participants. Analyses were conducted using an available-case approach. Descriptive statistics were calculated, and the Shapiro–Wilk test was employed to assess the normality of data distribution. Since most variables did not follow a normal distribution (Shapiro–Wilk test, *p* < 0.05), non-parametric tests were applied. Depending on distributional characteristics, the Mann–Whitney U test, one-sample Student’s *t*-test, Pearson’s correlation coefficient (*r*), and Spearman’s rank correlation coefficient (ρ) were applied. Multiple linear regression was employed to model the potential impact of predictors on a quantitative variable. The regression parameters, alongside the 95% confidence intervals, were presented. Variables included in the multivariate model were selected based on their statistical significance in univariate analyses. Given the limited sample size, this approach was intended to reduce model complexity. Multicollinearity was assessed using the Variance Inflation Factor (VIF), with values > 5 indicating potential multicollinearity. A significance threshold of α = 0.05 was adopted for all analyses.

Ethical approval for the study was obtained from the Bioethics Committee of Wrocław Medical University (decision no. KB 273/2023N; approved on 28 September 2023).

## 3. Results

The study population consisted of 100 patients with a mean age of 75.9 ± 9.8 years (range: 49–97). Men accounted for 63% of participants, and 60% resided in urban areas. More than half reported a monthly net household income > 2101 PLN (60%), indicating a relatively preserved socioeconomic status within the cohort (Table 1). The mean body weight was 83.4 ± 21.1 kg (range: 40–134 kg), with a mean BMI of 29.3 ± 6.5 kg/m^2^, indicating that the population was predominantly overweight. More than half of the participants reported 2–4 hospitalizations in the previous year (56%), and 30% had an implantable cardiac device. According to the NYHA functional classification, 41% were classified as class II and 57% as class III. Based on left ventricular ejection fraction (LVEF), 70% had HF with reduced ejection fraction (HFrEF; ≤40%), 22% had preserved ejection fraction (HFpEF; ≥50%), and 8% were classified as HFmrEF (41–49%) (Table 2).

The comparative analysis showed statistically significant differences between the study groups. NT-proBNP levels were significantly higher in patients who were malnourished or at risk of malnutrition compared to well-nourished individuals (*p* = 0.004). Triglyceride levels were significantly higher in well-nourished individuals (*p* = 0.032). NYHA class was higher in patients who were malnourished or at risk of malnutrition (*p* = 0.002) (Table 3).

The comparison of nutritional status between women and men, assessed using the Mini Nutritional Assessment (MNA) scale, revealed no statistically significant differences between the groups. The mean MNA scores were comparable in women (M = 19.9, SD = 5.0) and men (M = 19.9, SD = 4.9), with identical median values (Me = 21.5 in both groups). The Mann–Whitney U test indicated a non-significant difference (Z = −0.10, *p* = 0.917). Additionally, the effect size was negligible (r = 0.01; η^2^ < 0.01), suggesting no meaningful association between sex and nutritional status in the studied sample.

The self-care level of patients with chronic heart failure, assessed using the 9-item European Heart Failure Self-care Behaviour Scale (9-EHFScBS), was relatively low in the study group, with a mean score of 37.6 (SD = 16.3). The results of the one-sample Student’s t-test were statistically significant (t = −8.69, *p* < 0.001), with a 95% confidence interval ranging from −17.42 to −10.94.

**Figure 1:**
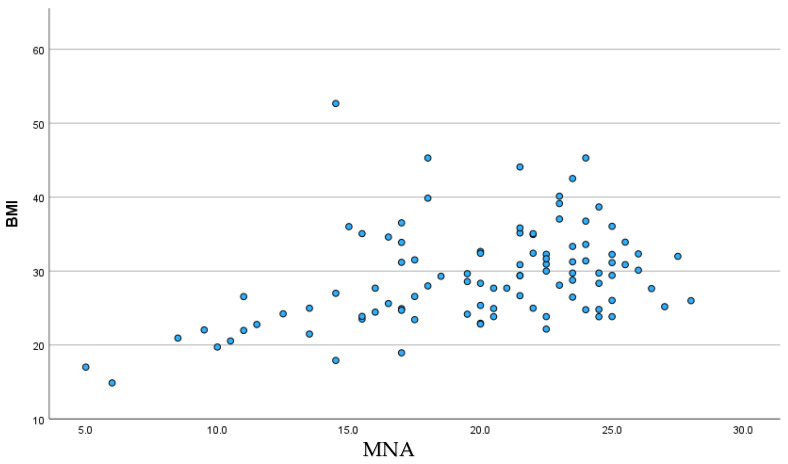
Correlation between body mass index (BMI) and nutritional status assessed with the Mini Nutritional Assessment (MNA) in patients with chronic heart failure.

**Figure 2:**
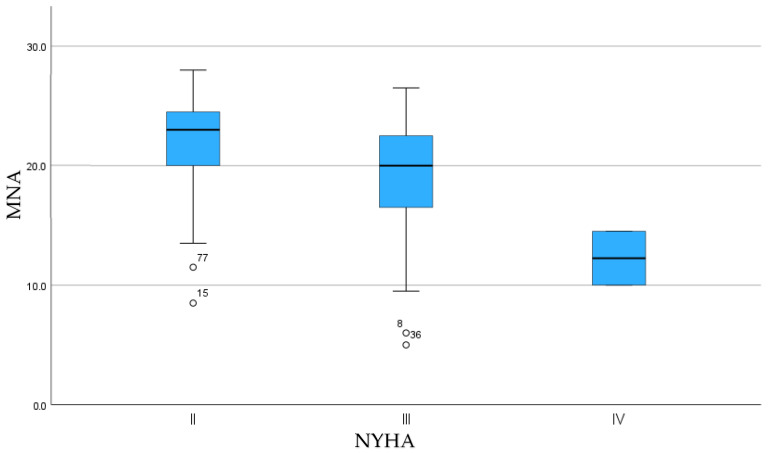
Distribution of nutritional status (MNA) across New York Heart Association (NYHA) functional classes in patients with chronic heart failure.

A significant positive correlation was found between BMI and nutritional status (r = 0.39; *p* < 0.001), suggesting that patients with higher BMI values had better nutritional status (Figure 1). Spearman’s rho correlation revealed a significant negative association between NYHA class and nutritional status (rho = −0.35; *p* < 0.001), indicating that more advanced HF was associated with poorer nutritional status (Figure 2).

Spearman’s rank correlation analysis was performed to examine the associations between NT-proBNP levels and selected nutritional parameters. A statistically significant, negative correlation was observed between NT-proBNP levels and nutritional status assessed using the MNA scale (rho = −0.27, *p* = 0.006). In contrast, no significant relationship was found between NT-proBNP levels and body mass index (BMI). Although the correlation coefficient was negative (rho = −0.10), the result did not reach statistical significance (*p* = 0.339).

Significant positive correlation was observed between MNA score and hemoglobin (r = 0.245, *p* = 0.014), hematocrit (r = 0.203, *p* = 0.043), albumin (r = 0.501, *p* = 0.048), and triglyceride levels (r = 0.314, *p* = 0.005). BMI showed significant negative correlations with total cholesterol (r = −0.317, *p* = 0.005), HDL cholesterol (r = −0.282, *p* = 0.013), and LDL cholesterol (r = −0.255, *p* = 0.025). Results are presented in Table 4.

Nutritional status was significantly and negatively correlated with the number of chronic comorbidities (r = −0.20; *p* = 0.045), indicating that a greater comorbidity burden was associated with poorer nutritional status. No significant associations were observed between nutritional status and the number of medications (r = 0.10; *p* = 0.324). Likewise, self-care level was not significantly related to either the number of comorbidities (r = −0.08; *p* = 0.415) or the number of medications (r = 0.10; *p* = 0.309). Finally, a weak but statistically significant positive correlation was observed between nutritional status and self-care level (r = 0.29; *p* = 0.003).

In further analysis, we performed a multivariate linear regression model. The model explained 44.0% of the variance in MNA score (R^2^ = 0.44), indicating a moderate model fit. No significant multicollinearity was observed among the predictors included in the model, as all VIF values were low (ranging from 1.096 to 1.177). The results showed that each additional point on the 9-EHFScBS increases the MNA score by an average of 0.083, each additional comorbidity decreases the MNA score by an average of 0.401, the NYHA III class lowers the MNA score by an average of 2.425 compared to NYHA II, and the NYHA IV class lowers the MNA score by an average of 5.966 compared to NYHA II. The analysis also showed that each additional kg/m^2^ of BMI raises the MNA score by an average of 0.368 (Table 5).

## 4. Discussion

Heart failure (HF) represents a significant global health challenge, marked by its chronic course and recurrent decompensations, which substantially impact patient prognosis and impose a considerable strain on healthcare systems [15]. In the 2022 AHA/ACC/HFSA guideline, lifestyle modification is emphasized as a cornerstone of HF care. The guideline also reinforces the importance of lifestyle-based prevention in earlier stages, including optimization of blood pressure, glycemic control, and adherence to a heart-healthy diet to delay or prevent the onset of overt HF [16]. Nutritional status plays a pivotal role in the clinical trajectory of HF, as both malnutrition and unintentional weight loss are associated with increased morbidity, rehospitalization, and mortality. The evaluation of nutritional status in HF patients is frequently neglected, reflecting the lack of uniform diagnostic criteria and reliable assessment instruments; moreover, current guidelines offer only minimal direction regarding the identification and treatment of nutritional disorders [8].

In patients with heart failure, nutritional disorders occur frequently, with prevalence estimates ranging from 16% to 90% [17]. Nutritional risk was common in the study cohort, consistent with previous reports. No significant differences in nutritional status were observed between female and male patients. Similar findings were reported by Kałużna-Oleksy et al., where half of patients with HF were identified as being at risk of malnutrition, yet no sex-related differences in nutritional status were demonstrated [18].

A significant association between NYHA functional class and nutritional status suggests that worsening functional status may contribute to nutritional deterioration. Patients with more advanced HF present with poorer nutritional status. These findings suggest that worsening functional class may contribute to nutritional deterioration and reinforce the need for nutritional screening in patients with advanced HF. Available research indicates that NYHA class was identified as an independent predictor of all-cause mortality, irrespective of sex and age [19]. Our results lend support to the notion that nutritional screening should be incorporated into standard risk stratification for heart failure, particularly in those with worse NYHA functional class.

The study by Pagnesi et al. demonstrated that lower BMI was independently associated with a higher risk of malnutrition in patients with severe heart failure [19]. This observation is consistent with our findings, in which a significant association between BMI and nutritional status was identified. However, the interpretation of BMI in patients with HF requires caution. BMI does not distinguish between fat mass, lean body mass, and fluid retention, which may influence its value in this population. In the present study, all patients were admitted electively and were clinically stable at the time of assessment, with no signs of acute heart failure decompensation. In addition, BMI was not significantly correlated with NT-proBNP levels, suggesting that it was not solely a marker of congestion severity. Nevertheless, BMI should be considered a simplified and indirect measure rather than a precise indicator of nutritional status. Its association with nutritional status should therefore be interpreted within the broader clinical context. Overall, BMI remains a readily available clinical parameter; however, due to the potential influence of fluid retention and altered body composition, it should be interpreted cautiously in patients with heart failure.

Notably, previous studies have shown that in multivariate regression analysis, among the clinical variables assessed, obesity was independently associated with a lower likelihood of prolonged hospital stay (OR = 0.205) [20]. Moreover, studies focusing on acute heart failure have shown that in well-nourished patients, the overweight or obese group had a lower risk of death in the short term compared to the group with a normal or below-normal BMI [21]. These findings support the concept that BMI, despite its limitations, may serve as a simple surrogate marker of nutritional status in patients with HF. Moreover, they are consistent with the so-called “obesity paradox,” which suggests that patients with higher BMI may experience better short-term outcomes [22]. Nonetheless, a comprehensive nutritional assessment remains essential, as BMI alone does not account for changes in body composition or fluid overload, both of which are highly prevalent in this population.

The observed association between NT-proBNP levels and nutritional status may reflect the link between worsening nutritional condition and greater disease severity.

Patients who were malnourished or at risk of malnutrition had significantly higher NT-proBNP levels, which is consistent with evidence indicating that poorer nutritional status is associated with greater neurohormonal activation and more advanced heart failure. A recent systematic review and meta-analysis reported that poorer nutritional status was significantly associated with higher levels of heart failure biomarkers, including BNP and NT-proBNP, as well as elevated inflammatory markers such as CRP [23]. These observations suggest that NT-proBNP might serve not only as a biomarker of cardiac stress and HF severity, but also as an indirect marker of systemic metabolic and nutritional deterioration. Higher NT-proBNP and a greater comorbidity burden may indicate more advanced disease, which could contribute to nutritional deterioration and highlight the need for comprehensive management.

A study by Ahmed et al. found that the presence of comorbidities was significantly associated with undernutrition in older adults with HF. Specifically, patients with severe HF, longer disease duration, and comorbidities were at increased risk of undernutrition [24]. We found a significant correlation between the number of chronic comorbidities and nutritional status. That aligns with existing literature, which consistently demonstrates that an increased comorbidity burden is associated with poorer nutritional status. These findings suggest that comprehensive management of HF should consider both the number and severity of comorbidities, as well as the patient’s nutritional status, to improve outcomes.

Effective self-care is essential for the management of heart failure (HF), and patients are encouraged to participate in structured self-care management programs. Self-care maintenance encompasses symptom monitoring, adherence to dietary recommendations, and compliance with prescribed medications, all of which contribute to maintaining a compensated clinical state [25]. Ensuring adequate nutrition is particularly important for optimizing clinical outcomes [26] and improving nutritional status may play a key role in enhancing self-care behaviors among HF patients. Available evidence indicates that structured nutritional management programs can effectively improve nutritional status in patients with HF [27]. In our study, a significant positive correlation was observed between nutritional status and self-care level, as assessed using the 9-EHFScBS questionnaire. The observed link between nutritional status and self-care behaviors suggests that improving nutrition may support patient engagement in self-management, although causal relationships cannot be determined in this cross-sectional study.

From a clinical perspective, the observed associations suggest that poorer nutritional status is linked to greater disease severity and may help identify patients at higher risk of adverse outcomes. In particular, the relationships with NYHA class and NT-proBNP indicate that nutritional deterioration may parallel heart failure progression. These findings support the importance of routine nutritional assessment as part of comprehensive heart failure management. Additionally, the association between nutritional status and self-care behaviours highlights the potential value of integrated clinical and behavioural interventions in this population.

## 5. Conclusions

This study demonstrated a relationship between nutritional status and clinical, biochemical, and behavioural factors in patients with chronic heart failure. Poorer nutritional status was associated with markers of greater disease severity, including higher NT-proBNP levels and a higher comorbidity burden. A modest association between nutritional status and self-care behaviours was also observed, suggesting a potential link between behavioural and clinical domains; however, this finding should be interpreted with caution due to the cross-sectional design. Although BMI was related to nutritional status, its clinical interpretation in heart failure remains limited. Overall, these findings support the need for a multidimensional approach to patient assessment and may support the integration of nutritional and behavioral assessment into routine clinical care in heart failure. Further longitudinal studies are warranted to clarify these relationships.

## 6. Limitations

This study has several limitations. First, due to its cross-sectional design, causal relationships between nutritional status, self-care behaviours, and clinical parameters cannot be established. Although patients were clinically stable, objective measures of volume status (e.g., bioimpedance analysis or standardized congestion scores) were not available. Therefore, residual confounding due to subclinical fluid retention cannot be completely excluded. Second, the study was conducted in a single tertiary centre and included a relatively small sample size, which may limit the external validity of the findings and reduce statistical power in subgroup analyses. Third, not all laboratory parameters were available for every participant, which may have introduced selection bias. For variables with smaller sample sizes (e.g., albumin), limited statistical power may have reduced the ability to detect differences between groups. Fourth, self-care behaviours were assessed using a self-reported instrument, which may be subject to recall and social desirability bias.

Therefore, the findings should be interpreted as exploratory and hypothesis-generating, and future longitudinal studies with larger, multicentre cohorts are warranted. Our findings may not apply to stable outpatient populations.

## Figures and Tables

**Table 1:** Sociodemographic Characteristics of the Study Group (*n* = 100).

Parameter	Total
Age [years]	
Mean ± SD	75.9 ± 9.8
Median	75.00
Min; Max	49; 97
Sex	
Female	37 (37%)
Male	63 (63%)
Place of Residence	
Rural	40 (40%)
Urban	60 (60%)
Occupational Status	
Unemployed	7 (7%)
Retired	88 (88%)
Employed	5 (5%)
Type of Work	
Manual	5 (5%)
White-collar	1 (1%)
Not applicable	94 (94%)
Marital Status	
Single	49 (49%)
In a relationship	51 (51%)
Education	
Primary	23 (23%)
Vocational	38 (38%)
Secondary	36 (36%)
Higher	3 (3%)
Net Household Income (PLN)	
601–1200	3 (3%)
1201–1800	6 (6%)
1801–2100	31 (31%)
>2101	60 (60%)

Abbreviations: SD—standard deviation; Min—minimum value; Max—maximum value; PLN—Polish zloty.

**Table 2:** Clinical Data Analysis of the Study Group (*n* = 100).

Parameter	Total
Weight [kg]	
Mean ± SD	83.4 ± 21.1
Median	80.00
Min; Max	40.00; 134.00
Height [m]	
Mean ± SD	1.68 ± 0.10
Median	1.69
Min; Max	1.30; 1.90
Body Mass Index [kg/m^2^]	
Mean ± SD	29.3 ± 6.5
Median	28.67
Min; Max	15.00; 53.00
Number of Hospitalizations in the Past Year	
1–2	40 (40%)
2–4	56 (56%)
>5	4 (4%)
Addictions	
No	71 (71%)
Yes	29 (29%)
Implantable Device	
No	70 (70%)
Yes	30 (30%)
NYHA Class	
I	0 (0%)
II	41 (41%)
III	57 (57%)
IV	2 (2%)
HF Classification Based on LVEF (%)	
≤40% (HFrEF)	70 (70%)
41–49% (HFmrEF)	8 (8%)
≥50% (HFpEF)	22 (22%)

Abbreviations: SD—standard deviation; Min—minimum value; Max—maximum value; NYHA—New York Heart Association functional class; LVEF—left ventricular ejection fraction; HFrEF—heart failure with reduced ejection fraction; HFmrEF—heart failure with mildly reduced ejection fraction; HFpEF—heart failure with preserved ejection fraction.

**Table 3:** Comparison of clinical variables between groups depending on nutritional status.

Parameter		Malnutrition/Risk of Malnutrition (*n* = 76)	Proper Nutritional Status (*n* = 24)	*p*
NT-proBNP [1000 pg/mL]	Median (quartiles)	7.61 (3.3–14.62)	3.14 (1.49–5.23)	*p* = 0.004 *
	Range	1.04–62.53	0.91–29.4	
	*n*	76	24	
WBC [×10^3^/µL]	Median (quartiles)	7.69 (5.89–10.13)	7.15 (5.79–8.88)	*p* = 0.358
	Range	2.17–19.61	3.82–11.28	
	*n*	76	24	
RBC [×10^6^/µL]	Median (quartiles)	4.11 (3.61–4.62)	4.23 (3.79–4.46)	*p* = 0.553
	Range	2.78–5.67	2.85–5.3	
	*n*	76	24	
Hgb [g/dL]	Median (quartiles)	12.15 (10.65–13.53)	13.3 (10.83–14.93)	*p* = 0.096
	Range	8.1–16.7	8.3–16	
	*n*	76	24	
Ht [%]	Median (quartiles)	35.85 (32.55–40.45)	38.2 (32.67–42.92)	*p* = 0.211
	Range	22.8–49.4	26.2–47.8	
	*n*	76	24	
PLT [×10^3^/µL]	Median (quartiles)	209.5 (151.75–274)	195 (176–242)	*p* = 0.801
	Range	84–898	87–392	
	*n*	74	24	
Alb [g/dL]	Median (quartiles)	3.36 (3.19–3.6)	3.68 (3.66–3.86)	*p* = 0.189
	Range	2.2–4.5	3.65–4.03	
	*n*	13	3	
TC [mg/dL]	Median (quartiles)	133 (112–160)	133 (122–169)	*p* = 0.506
	Range	75–234	98–203	
	*n*	61	17	
HDL [mg/dL]	Median (quartiles)	43.9 (35–52.9)	42 (37–52.9)	*p* = 0.758
	Range	20–116	22–82	
	*n*	61	17	
non-HDL [mg/dL]	Median (quartiles)	94 (66.03–115.5)	100 (73–115)	*p* = 0.539
	Range	38–202	40–156	
	*n*	60	17	
LDL [mg/dL]	Median (quartiles)	67 (48–91)	68 (51.91–87)	*p* = 0.846
	Range	25–165	33.54–115	
	*n*	61	16	
TG [mg/dL]	Median (quartiles)	99 (75–132)	148 (97–201)	*p* = 0.032 *
	Range	48–276	63–321	
	*n*	61	17	
NYHA	NYHA II	24 (31.58%)	17 (70.83%)	*p* = 0.002 *
	NYHA III	50 (65.79%)	7 (29.17%)	
	NYHA IV	2 (2.63%)	0 (0.00%)	
BMI [kg/m^2^]	Median (quartiles)	28.04 (24.09–32.47)	30.49 (26.02–32.64)	*p* = 0.157
	Range	14.87–52.66	23.82–45.29	
	*n*	76	24	
LVEF [%]	Median (quartiles)	35 (25–45)	40 (25–47)	*p* = 0.74
	Range	14–60	16–60	
	*n*	76	24	

Abbreviations: *p*—Qualitative variables: chi-squared or Fisher’s exact test. Quantitative variables: Mann–Whitney test, * statistically significant (*p* < 0.05).

**Table 4:** Results of Spearman’s correlation between Nutritional Status (MNA), BMI and Selected Laboratory Parameters.

Variable	MNA	BMI
White blood cells (×10^3^/µL)	r = −0.071 *p* = 0.484	r = −0.079 *p* = 0.432
Red blood cells (×10^6^/µL)	r = 0.124 *p* = 0.219	r = 0.077 *p* = 0.449
Hemoglobin (g/dL)	r = 0.245 *p* = 0.014 *	r = −0.074 *p* = 0.464
Hematocrit (%)	r = 0.203 *p* = 0.043 *	r = 0.026 *p* = 0.794
Platelets (×10^3^/µL)	r = −0.045 *p* = 0.657	r = 0.078 *p* = 0.448
Albumin (g/dL)	r = 0.501 *p* = 0.048 *	r = 0.371 *p* = 0.158
Total cholesterol (mg/dL)	r = 0.046 *p* = 0.692	r = −0.317 *p* = 0.005 *
HDL cholesterol (mg/dL)	r = −0.043 *p* = 0.711	r = −0.282 *p* = 0.013 *
Non-HDL cholesterol (mg/dL)	r = 0.077 *p* = 0.505	r = −0.176 *p* = 0.126
LDL cholesterol (mg/dL)	r = 0.022 *p* = 0.848	r = −0.255 *p* = 0.025 *
Triglycerides (mg/dL)	r = 0.314 *p* = 0.005 *	r = 0.209 *p* = 0.066

r—Spearman’s correlation coefficient. * statistically significant (*p* < 0.05).

**Table 5:** Multivariate linear regression model.

Independent Variable		B	95%CI		*p*
9-EHFScBS		0.083	0.032	0.133	0.002 *
NT-proBNP [1000 pg/mL]		0.009	−0.055	0.074	0.775
Hgb [g/dL]		0.38	−0.007	0.767	0.054
Number of chronic comorbidities		−0.401	−0.766	−0.036	0.032 *
NYHA	NYHA II	ref.			
	NYHA III	−2.425	−4.053	−0.797	0.004 *
	NYHA IV	−5.966	−11.7	−0.233	0.042 *
BMI [kg/m^2^]		0.368	0.246	0.49	<0.001 *

*p*—multiple linear regression. * statistically significant (*p* < 0.05).

## Data Availability

The datasets generated and analyzed during the current study are available from the corresponding author on reasonable request. Due to ethical and privacy restrictions related to patient data, the datasets are not publicly available.
